# Nanoscale Hydrophobicity of Transport Barriers in the Nuclear Pore Complex as Compared with the Liquid/Liquid Interface by Scanning Electrochemical Microscopy

**DOI:** 10.1021/acs.analchem.4c04861

**Published:** 2025-01-29

**Authors:** Siao-Han Huang, Moghitha Parandhaman, Manu Jyothi Ravi, Donald C. Janda, Shigeru Amemiya

**Affiliations:** Department of Chemistry, University of Pittsburgh, 219 Parkman Avenue, Pittsburgh, Pennsylvania 15260, United States

## Abstract

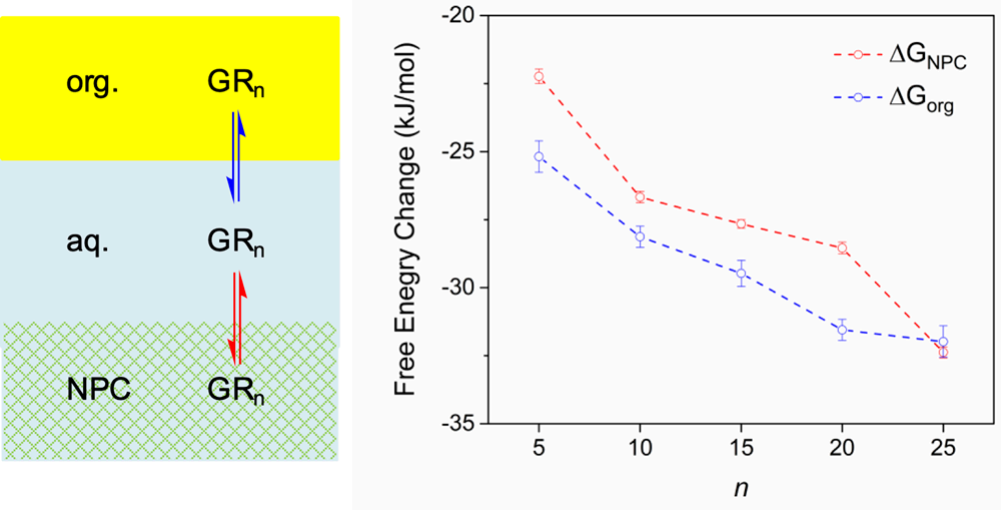

The nuclear pore complex (NPC) is the proteinous nanopore that solely regulates molecular transport between the nucleus and cytoplasm of a eukaryotic cell. Hypothetically, the NPC utilizes the hydrophobic barriers based on the repeats of phenylalanine–glycine (FG) units to selectively and efficiently transport macromolecules. Herein, we quantitatively assess the hydrophobicity of transport barriers confined in the nanopore by applying scanning electrochemical microscopy (SECM). The hypothesis deduced from studies of isolated FG-rich nucleoporins is supported quantitatively by investigating the authentic NPC for the first time. Specifically, we employ the *n* repeats of neurotoxic glycine–arginine dipeptide, GR_*n*_, as the molecular probes that engage in hydrophobic interactions with transport barriers in the NPC. We apply ion-transfer voltammetry at a micropipet-supported interface between aqueous and organic electrolyte solutions to confirm that larger GR_*n*_ among *n* = 5–25 is more hydrophobic, as expected theoretically. The micropipet also serves as the tip of transient SECM to demonstrate that the NPC interacts more strongly with larger GR_*n*_, which supports the hydrophobicity of transport barriers. Kinetically, larger GR_*n*_ stays in the NPC for longer to clog the nanopore, thereby expressing neurotoxicity. Significantly, this work implies that the efficient and safe nuclear import of genetic therapeutics requires an optimum balance between strong association with and fast dissociation from the NPC. Interestingly, this work represents the unexplored utility of liquid/liquid interfaces as models of hydrophobic protein condensates based on liquid–liquid phase separation as exemplified by nanoscale transport barriers in the NPC.

The nuclear pore complex (NPC) solely and selectively mediates molecular transport between the nucleus and cytoplasm of eukaryotic cells to play imperative biological^1,2^ and biomedical^3^ roles. The selective transport of macromolecules through the NPC has been attributed to the hydrophobic transport barriers that are rich in the repeats of the phenylalanine–glycine (FG) unit. Hydrophobic interactions among FG units drive liquid–liquid phase separation^4^ (LLPS) to form mesh-like barriers.^5^ The meshes are small enough^6^ to prevent the passive transport of large macromolecules.^7^ A passively impermeable macromolecule can be tagged with the nuclear localization signal, which is recognized by a nuclear transport receptor, e.g., importin.^8^ The signal-dependent macromolecular transport is facilitated by hydrophobic interactions of importin with transport barriers. Hydrophobic FG-rich nucleoporins (nups) are crucial also for RNA export by exportins^9^ and can be blocked by SARS-CoV-2 Orf6 to initiate viral infections.^10^ Moreover, HIV-1 capsids utilize hydrophobic interactions with FG-rich nups to enter the nucleus.^11^ The importance of hydrophobic interactions, however, has been assessed by studying the hydrogels of natural^12^ and mutated^13^ FG-rich nups and synthetic analogs.^14−16^

Herein, we employ dipeptide repeats of glycine and arginine, GR_*n*_ (Figure 1A), as molecular probes to examine the hydrophobicity of nanoscale transport barriers as confined in the authentic NPC. Dipeptide repeats of GR are contained in the aggregation-prone proteins that originate from the disordered *C9orf72* gene to cause such serious neurological diseases as amyotrophic lateral sclerosis and frontotemporal dementia.^17,18^ Recent molecular dynamics studies confirmed that GR_25_ is more hydrophobic than GR_12_^19,20^ as expected from the strong propensity of arginine-containing dipeptide repeats for LLPS.^21^ In this work, we find that larger GR_*n*_ associates with the NPC more strongly (Figure 1B), which supports the hypothesis that the transport barriers based on FG-rich nups are hydrophobic.^12,13^ In addition, we reveal that larger GR_*n*_ stays in the NPC for longer to clog the nanopore as the cause of neurotoxicity. The thermodynamic and kinetic insights obtained in this work are highly significant biomedically. Macromolecular and nanomaterial therapeutics for genetic diseases^3^ must not only strongly associate with but also rapidly dissociate from the NPC to enter the nucleus efficiently and safely.

**Figure 1:**
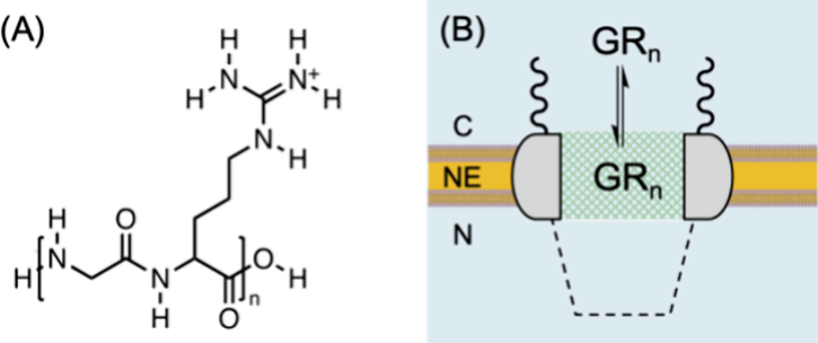
Scheme of (A) GR_*n*_ (*n* = 5, 10, 15, 20, and 25) and (B) its transfer from the aqueous solution to the hydrophobic mesh-like transport barrier (green) in the NPC (gray) with cytoplasmic filaments (wavy line) and a nuclear basket (dotted line). C and N are the cytoplasmic and nucleoplasmic sides, respectively. Lipid bilayers are the outer and inner nucleus membranes of the nuclear envelope (NE).

Specifically, we apply the transient mode of scanning electrochemical microscopy^22^ (SECM) to measure the thermodynamics and kinetics of NPC–GR_*n*_ interactions. We developed transient SECM to demonstrate similar hydrophobic interactions of the NPC with GR_20_, protamine, and 20 repeats of proline and arginine, PR_20_.^23^ The nuclear envelope (NE) is isolated from the nucleus of a *Xenopus laevis* oocyte to spread over a microporous Si_3_N_4_ membrane (Figure 2).^24,25^ The micropore-supported NE is equilibrated with GR_*n*_, which is associated with the NPCs. The association equilibrium is disturbed by the micropipet doped with the nitrobenzene (NB) solution of an ionophore, dinonylnaphthalenesulfonate (DNNS). GR_*n*_ is depleted near the micropipet tip to dissociate from the NPCs and diffuse to the tip. The transient enhancement of the tip current is analyzed to determine the strength and kinetics of NPC–GR_*n*_ interactions and the concentration of interaction sites,^22^ i.e., FG units.^23^ This measurement is biologically relevant because the NPCs of the micropore-supported NE mediate macromolecular transport as expected physiologically.^26^ The organic solvent leached from a micropipet does not affect the NPC permeability as measured with different solvents^25^ and Pt tips.^27,28^

**Figure 2:**
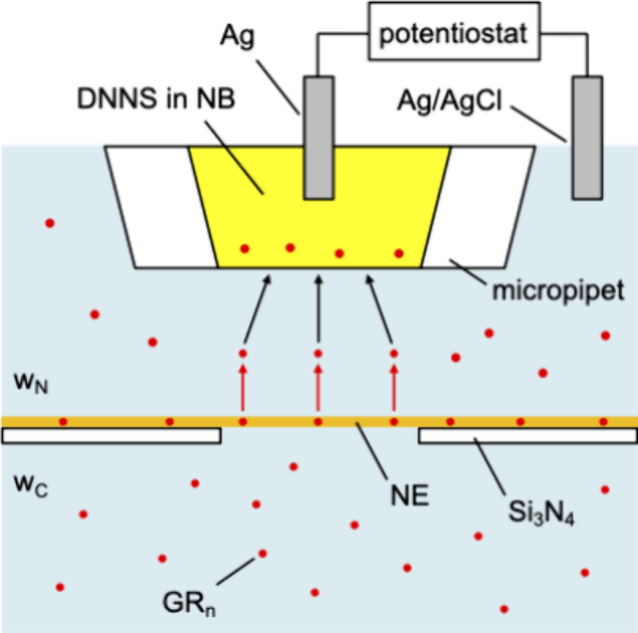
Transient SECM measurement of NPC–GR_*n*_ interactions in the NE supported by a microporous Si_3_N_4_ membrane. Aqueous solutions at the cytoplasmic and nucleus sides of the NE are indicated by w_C_ and w_N_, respectively. Red and black arrows indicate the dissociation and diffusion of GR_*n*_, respectively.

Interestingly, this work is the first to propose a liquid/liquid interface as a model of hydrophobic proteinous environments based on LLPS,^29^ as exemplified by nanoscale transport barriers in the NPC.^4^ We reveal that larger GR_*n*_ is transferred more favorably not only into the NPC but also into the hydrophobic NB solution (Figure 2). This agreement is highly quantitative as supported by similar free energy changes in the respective transfer processes, Δ*G*_NPC_ and Δ*G*_org_. The similar dependence of Δ*G*_NPC_ and Δ*G*_org_ on the size of GR_*n*_ is attributed to the similar hydrophobicity of the two environments. The polarity of the water-saturated NB solution (ε_r_ = 35.5^30^) is similar to that of LLPS-based protein condensates (ε_r_ = 30–60^31^). Previously, a liquid/liquid interface was considered as a model of biological membranes^32^ despite much lower polarity (ε_r_ = ∼3^33^). Experimentally, transient SECM yields the equilibrium constant of NE–GR_*n*_ association, β, which is used to calculate Δ*G*_NPC_. We employ ion-transfer cyclic voltammetry at the micropipet-supported liquid/liquid interface^34,35^ to determine Δ*G*_org_. The Δ*G*_org_ value is estimated for each GR_*n*_ from the half-wave potential, *E*_1/2_, based on an extra-thermodynamic assumption.^36^

## Experimental Section

### Chemicals and Materials

GR_*n*_ was synthesized at the Peptide and Peptoid Synthesis Core of the University of Pittsburgh by using standard FMOC chemistry cycles on a Liberty Blue microwave synthesizer (CEM, Matthews, North Carolina), purified by HPLC on a Luna C18 column (Phenomenex, Torrance, California), and characterized by MALDI-TOF MS (Bruker, Billerica, Massachusetts) for the final confirmation of the expected target mass. Poly(vinylpyrrolidone) (PVP; average molecular weight, 40 kDa), NB (≥99%), tetradodecylammonium (TDDA) bromide, and chlorotrimethylsilane (≥99%) were purchased from Sigma-Aldrich (St. Louis, Missouri). Potassium tetrakis(pentafluorophenyl)borate (TFAB) was obtained from Boulder Scientific (Mead, Colorado). Dinonylnaphthalene sulfonic acid (Nacure 1052) was a gift from King Industries (Norwalk, Connecticut). The TDDA salts of DNNS^37^ and TFAB^38^ were prepared by metathesis. Silicon nitride (Si_3_N_4_) membranes with a 200 nm-thick squared microporous region with 10 μm in pore diameter and 1.8 mm in length (NX5200DH10) were obtained from Norcada (Edmonton, Canada). A Milli-Q IQ 7003 water purification system (EMD Millipore, Billerica, Massachusetts) was used to obtain UV-treated deionized ultrapure water (18.2 MΩ·cm) with total organic carbon of 2–3 ppb.

### Micropore-Supported NE

The nucleus was isolated from the stage VI oocyte of an adult female *X. laevis* frog (Ecocyte Bioscience, Austin, Texas) to spread the NE on a microporous Si_3_N_4_ membrane, as reported elsewhere.^24^ The large nucleus (∼0.4 mm in diameter) was isolated in the isotonic 1.5% PVP solution of mock intracellular buffer (MIB) at pH 7.4. MIB contains 90 mM KCl, 10 mM NaCl, 2 mM MgCl_2_, 1.1 mM EGTA, 0.15 mM CaCl_2_, and 10 mM HEPES, where free Ca^2+^ was buffered at the physiological level of ∼200 nM in oocytes.^39^ The spread NE was adhered to the membrane treated with Cell-Tak (Corning, Corning, New York).

### SECM

A home-built SECM instrument^40^ with a potentiostat (CHI 7042E, CH Instruments, Austin, Texas) was controlled by using a custom LabVIEW program (National Instruments, Austin, Texas).^41^ A micropore-supported NE was set up in the SECM cell, as shown in Figure S1. Tapered micropipets were obtained from borosilicate glass capillaries (o.d./i.d. = 1.0 mm/0.58 mm, 10 cm in length) using a CO_2_-laser capillary puller (model P-2000 Sutter Instrument, Novato, California). A program of heat = 470, filament = 4, velocity = 25, delay = 140, and pull = 0 was repeated four times to yield a pair of micropipets. The tapered end of a micropipet was milled by the focused ion beam (FIB) of Ga^+^ using a DualBeam instrument (Scios, FEI, Hillsboro, Oregon), as reported elsewhere.^23,42,43^ The entire surface of FIB-milled glass micropipets was modified with chlorotrimethylsilane in a vacuum-dried desiccator.^44^ The hydrophobic tips were filled with the NB solution of 40 mM TDDADNNS and 100 mM TDDATFAB and equipped with an Ag electrode (671440, A-M Systems, Sequim, Washington). The NB solution was not saturated or contacted with water until the micropipet tip was immersed in the MIB solution.

## Results and Discussion

### Voltammetric Hydrophobicity of GR_*n*_ at the Liquid/Liquid Microinterface

**Figure 3:**
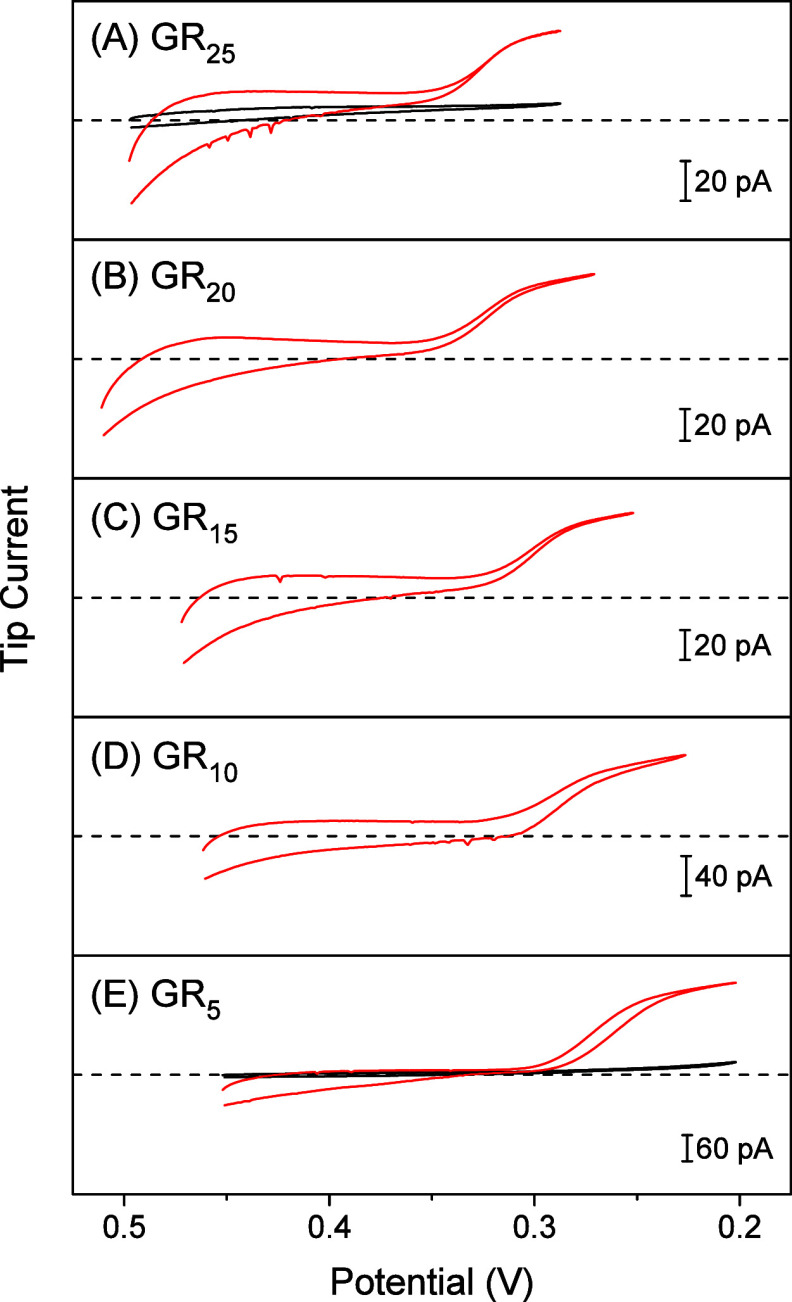
Cyclic voltammograms of GR_*n*_ (red lines) as transferred across the NB/water interface supported by 10 μm-diameter DNNS-based micropipets in Tris/acetate buffer at pH 7.8. Scan rate, 10 mV/s. The concentrations of (A) GR_25_, (B) GR_20_, (C) GR_15_, (D) GR_10_, and (E) GR_5_ are 8.6, 10, 14, 21, and 130 μM, respectively. Black solid lines represent background cyclic voltammograms. Dashed lines represent zero current. The potential of the NB phase against the aqueous phase was defined by using an extra-thermodynamic assumption to yield a formal potential of 0.29 V for tetrabutylammonium transfer (see the main text).

We employed steady-state cyclic voltammetry at micropipe-supported liquid/liquid interfaces (red lines in Figure 3) to evaluate the hydrophobicity of GR_*n*_. A micropipet was filled with the NB solution of a negatively charged ionophore, DNNS, as a TDDA salt in addition to TDDATFAB as supporting electrolytes. The pipet was immersed in the Tris/acetate buffer solution of GR_*n*_ at pH 7.8 to measure cyclic voltammograms in the wide range of the potential.^45^ The interfacial potential was controlled by biasing the Ag electrode in the NB solution against the Ag/AgCl electrode in the aqueous solution (Figure 2). The potential was calibrated against the formal potential of tetrabutylammonium transfer, as detailed below. Reproducible voltammograms are obtained by smoothing the tip end of a pulled micropipet using FIB, thereby forming a stable liquid/liquid interface.^42^ Background cyclic voltammograms in the Tris/acetate buffer (black solid lines in Figure 3A,E) confirm selective GR_*n*_ transfer in this potential range. GR_*n*_ transfer was also selectively detected when the aqueous solution contained physiological ions for SECM experiments with the NE.^23^

Well-defined steady-state cyclic voltammograms were obtained at the scan rate of 10 mV/s to require less negative potentials for higher GR_*n*_ with lower *n* (red lines in Figure 3). This result indicates that the transfer of larger GR_*n*_ is more favorable, which is attributed to the higher hydrophobicity of larger GR_n_, as predicted theoretically.^19,20^ The position of a cyclic voltammogram was quantitatively evaluated by the half-wave potential, *E*_1/2_, where the tip current during the forward potential sweep increased to half of the diffusion-limited current response of the micropipet in the bulk solution, *i*_T,∞_, as given by
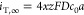
1where *x* is a function of RG^46^ (= *r*_g_/*a* = 1.4 for *x* = 1.18; *a* = 5 μm and *r*_g_ = 7 μm are the inner and outer radii of a micropipet tip, respectively, and were determined by scanning electron microscopy^23^), *z* (= +*n*) is the charge of the polypeptides, *D* (= 1.2 × 10^–6^ cm^2^/s) is the diffusion coefficient of GR_*n*_ as estimated by chronoamperometry for protamine with similar size to GR_*n*_,^47^*F* is the Faraday constant, and *c*_0_ is the bulk concentration of GR_*n*_. The *E*_1/2_ value of larger GR_*n*_ is more positive (Table 1), which corresponds to a more favorable transfer into the NB phase.

**Table 1:** Half-Wave Potential of GR_*n*_ at the NB/Water Interface and Parameters for Interactions of GR_*n*_ with the NPC^a^

		homogeneous model^c^			heterogeneous model^c^		
*n*	*E*_1/2_ (V)^b^	β (1/Μ)	*k*_diss_ (1/s)	Γ_S_ (pmol/cm^2^)	*k*_diss,NPC_ (1/s)	Γ_S,NPC_ (nmol/cm^2^)	*N*_p_ (molecule)
25	0.332 ± 0.006	(4.7 ± 0.9) × 10^5^	2.1 ± 0.8	7.4 ± 0.9	0.09 ± 0.03	1.0 ± 0.1	(1.1 ± 1) × 10^4^
20	0.327 ± 0.004	(1.0 ± 0.2) × 10^5^	7 ± 1	6.9 ± 0.5	0.30 ± 0.04	0.96 ± 0.06	(1.04 ± 0.07) × 10^4^
15	0.306 ± 0.005	(7 ± 1) × 10^4^	7 ± 3	7 ± 1	0.3 ± 0.1	0.9 ± 0.1	(1.0 ± 1) × 10^4^
10	0.292 ± 0.004	(4.7 ± 0.9) × 10^4^	10 ± 5	10 ± 4	0.5 ± 0.2	1.3 ± 0.5	(1.4 ± 5) × 10^4^
5	0.261 ± 0.006	(8 ± 2) × 10^3^	30 ± 20	18 ± 5	1.6 ± 0.8	2.4 ± 0.7	(2.6 ± 8) × 10^4^

a Models and parameters are defined in the main text and Supporting Information.

b *N* = 3.

c *N* = 5, 7, 6, 6, and 6 for *n* = 25, 20, 15, 10, and 5, respectively.

We calibrated the potential of the NB phase against the aqueous phase by using an extra-thermodynamic assumption (Figure 3) to calculate the free energy change in GR_*n*_ transfer, as discussed later. Specifically, it was assumed that the free energies of tetraphenylarsonium and tetraphenylborate transfer across the interface are identical.^36^ This assumption yields a formal potential of 0.29 V for tetrabutylammonium transfer across the NB/water interface.^48^ Half-wave and formal potentials are identical for electrochemically reversible transfer of tetrabutylammonium.

It should be noted that cyclic voltammograms of GR_*n*_ transfer (Figure 3) are electrochemically irreversible, thereby yielding steady-state responses during both forward and reverse scans, as detailed elsewhere.^45^ Briefly, a micropipet tip is small enough to achieve the steady-state diffusion of GR_*n*_ in the aqueous solution, as demonstrated by the sigmoidal positive current response when the interfacial potential is negative enough to drive ingressive GR_*n*_ transfer from the aqueous phase into the NB phase during forward and reverse scans. By contrast, the pipet wall hinders the diffusion of the GR_*n*_–DNNS complex in the NB phase during the reverse scan to anticipate a negative peak current response based on regressive GR_n_ transfer from the NB phase to the aqueous phase. A negative peak current response, however, was not observed within the potential window during the reverse scan, which indicates that GR_*n*_ transfer is electrochemically irreversible.

### Steady-State SECM Measurement of NPC Permeability to GR_*n*_

We employed DNNS-doped micropipets to measure the similar steady-state permeability of the NPCs to GR_*n*_. In steady-state SECM measurements, the potential of a micropipet was set to negative enough to yield the current limited by the diffusion of GR_*n*_ from the aqueous solution to the tip. Here, GR_25_ and GR_5_ are represented not only to illustrate the steady-state measurement of similar permeability but also to reveal the presence and absence of strong transient responses to the respective polypeptides. The strong transient response to GR_25_ is attributed to the dissociation of GR_25_ from the NPC as induced by an SECM tip and was investigated quantitatively by chronoamperometry below to confirm strong NPC–GR_25_ interactions. Both steady-state permeability measurement and chronoamperometry were performed by positioning a micropipet tip over the center of the NE patch supported by a micropore. SECM imaging identified the center of the micropore-supported NE patch. The resultant axisymmetry of the tip–NE configuration simplified the finite element analysis of SECM results.^23^ Moreover, the identical 10 μm diameter of micropipets and micropores facilitated data analysis by observing transient dissociation of GR_*n*_ only from the NE patch supported by the micropore.

**Figure 4:**
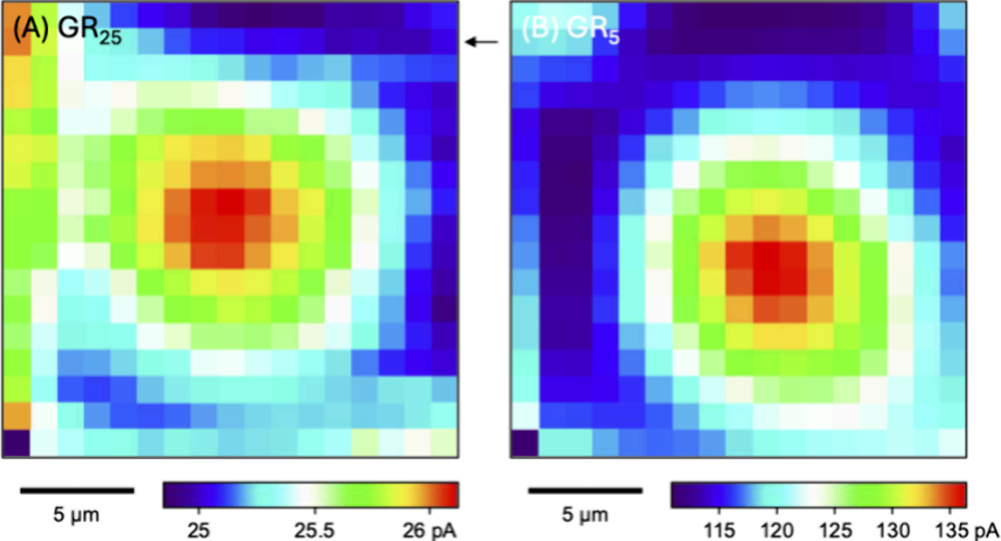
SECM images of self-standing NE patches supported by a 10 μm-diameter micropore as obtained by measuring the diffusion-limited current response of 10 μm-diameter micropipets to (A) 8.6 μM GR_25_ or (B) 130 μM GR_5_ in MIB. The tip was stepped by 1.25 μm every 2 s. Tip–NE distances of (A) 16.5 and (B) 16.0 μm were determined by the analysis of approach curves (see Figure 5). The arrow in part (A) indicates the line scan that confirms the flatness and negligible tilt of the substrate.

SECM imaging was employed to locate the center of the NE patch supported by a micropore. The current response of a micropipet to GR_25_ was enhanced when the tip was scanned over the micropore-supported NE (Figure 4A). This result ensures that GR_25_ is transported across the self-standing NE from the bottom solution to the top solution and then detected at the tip (Figure 2). Similarly, GR_5_ was transported across the micropore-supported NE, where the tip current response was enhanced (Figure 4B). SECM images, however, looked different between GR_25_ and GR_5_ on the left-hand side. The current response of a micropipet to GR_25_ was transiently enhanced when the tip was suddenly moved from the right-hand side to the left-hand side for the next line scan. The micropipet was suddenly exposed to the fresh solution of GR_25_ on the left-hand side to induce the dissociation of GR_25_ from the NPC. The transient response was observed reproducibly and was also reported for GR_20_.^23^ Moreover, the transient enhancement of the tip current response to GR_25_ is not due to the tilt of the substrate. The SECM line scan of a region surrounding the NE patch confirms (indicated by the arrow in Figure 4A) that the tip current increased transiently but decayed quickly to stay constant at the right-hand side of the substrate, which is flat and negligibly tilted. By contrast, the transient response was not observed with GR_5_, which interacts with the NPC but much more weakly (see below).

**Figure 5:**
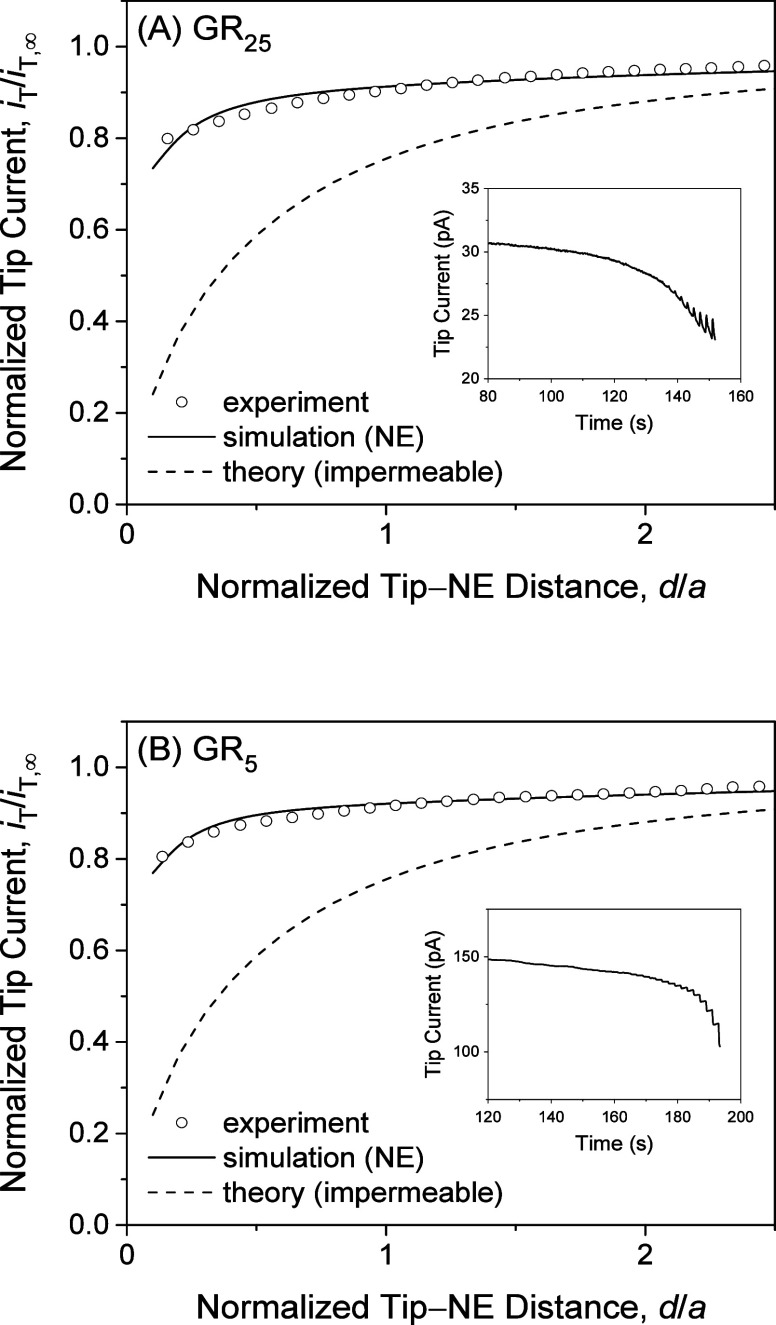
Experimental and simulated approach curves at the center of the micropore-supported NE patches as obtained by measuring the diffusion-limited current response of 10 μm-diameter micropipets to (A) 8.6 μM GR_25_ or (B) 130 μM GR_5_ in MIB. Simulation employed *k*_ss_ = 7.2 × 10^–3^ and 1.1 × 10^–2^ cm/s, respectively. The inset shows the time profile of the tip current with the sampling interval of 0.1 s during an approach to the NE with a 0.5 μm step every 2 s.

The similar steady-state permeability of the NPC to GR_25_ and GR_5_ was determined by employing approach curves. As the tip approached the NE, the tip current decreased gradually because the inner and outer membranes of the NE hindered the diffusion of the polypeptides to the tip. The plot of the tip current versus the vertical tip position from the NE was compared with the theoretical curve based on the finite element simulation,^23^ which utilized the homogeneous model, as discussed below. We employed COMSOL Multiphysics to perform the finite element simulation, as reported elsewhere^23^ (also, see the Supporting Information). A good fit was obtained for GR_25_ and GR_5_ (Figure 5) to yield similar steady-state permeability, *k*_ss_, of 7.2 × 10^–3^ and 1.1 × 10^–2^ cm/s, respectively, through the NE. The *k*_ss_ values are consistent with the diameter, length, and density of the NPCs,^25^ thereby excluding a transport pathway through inner and outer membranes. The difference between the *k*_ss_ values is comparable to uncertainty in the analysis of an experimental approach curve, which can be fitted with simulated curves when the permeability is changed by ±20% in the simulation.^27^ The approach curves for GR_25_ and GR_5_ were plotted against the tip–NE distance, *d*, as normalized against the tip radius, *a*, to resemble each other. By contrast, the experimental time profile of the tip current was recorded during the tip approach to manifest transient responses to GR_25_ but not to GR_5_. The current response to GR_25_ increased transiently whenever the tip stepped near the NE (*t* > 140 s in the inset of Figure 5A). The transient current response is attributed to the dissociation of GR_25_ from the NE, which was induced by the closer positioning of the micropipet tip to the NE. A transient response to GR_5_ was not observed during the tip approach (inset in Figure 5B), which indicates weak NPC–GR_5_ interactions as confirmed by chronoamperometry below.

It should be noted that the permeability of the NPC was not affected by the organic solvent leached from a micropipet during SECM measurements.^28^ The *k*_ss_ values of GR_25_ and GR_5_ were obtained by filling micropipets with the NB solution and are consistent with the theoretical values validated for small ions and polyions by filling micropipets with 1,2-dichloroethane (DCE) and NB solutions, respectively (see Figure 6 of ref (25)). Moreover, the NPC demonstrated the theoretically expected permeability to (ferrocenylmethyl)trimethylammonium as measured by either Pt tips^24,27^ or micropipets filled with the 1,2-DCE solution.^25,43^

**Figure 6:**
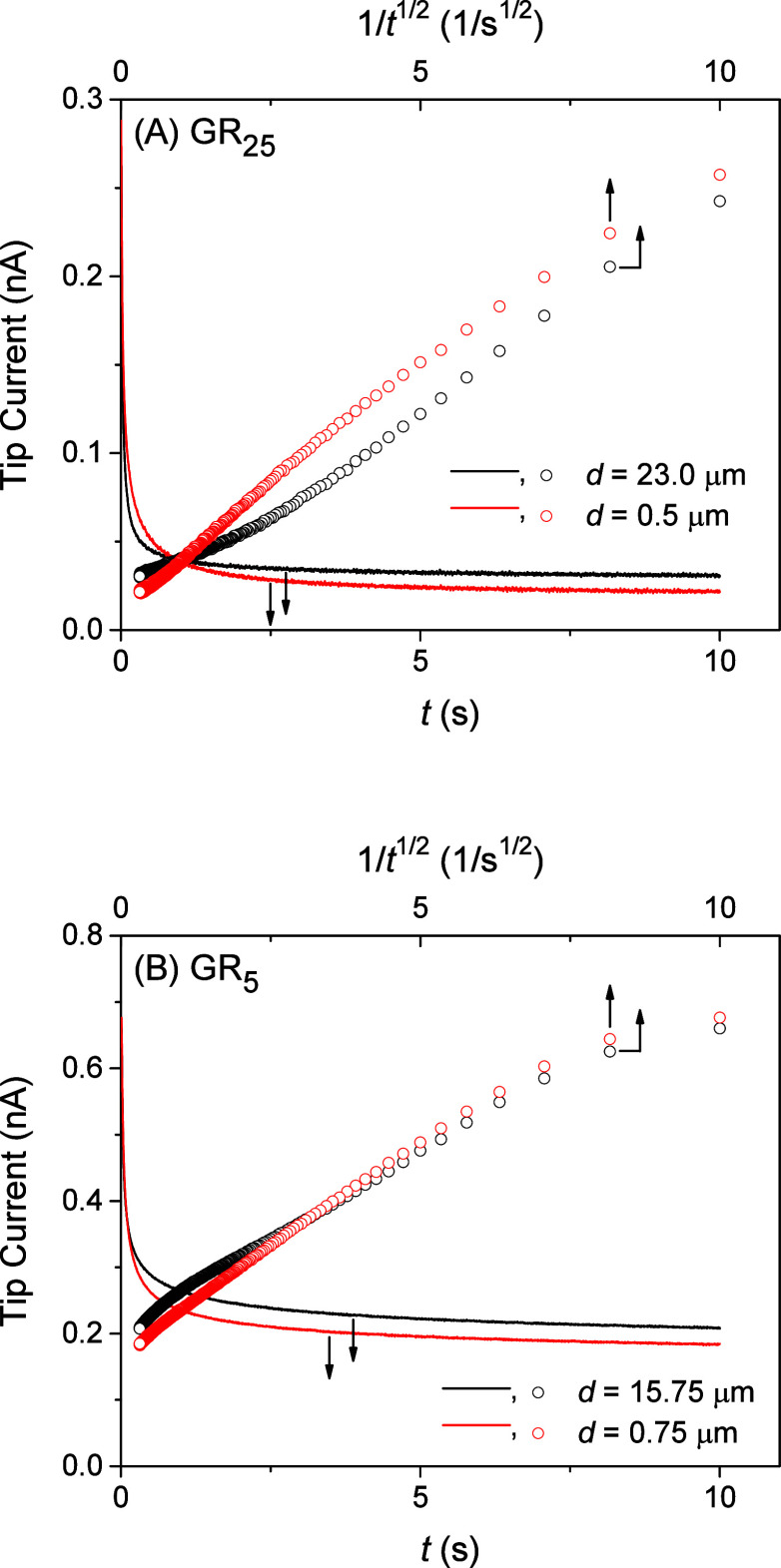
Chronoampergrams of (A) 8.6 μM GR_25_ or (B) 130 μM GR_5_ at 10 μm-diameter micropipet tips positioned far from (black) and near (red) the NE in MIB. Sampling interval, 5 ms. The tip–NE distance, *d*, was determined by the analysis of the chronoamperograms in Figure 7.

### Transient SECM Measurement of NE–GR_*n*_ Interactions

We employed the transient mode of SECM to quantitatively evaluate interactions of the NE with GR_*n*_. The interactions were modeled by the Langmuir-type isotherm to determine thermodynamic and kinetic parameters.^23^ There is a rate constant for dissociation of GR_*n*_ from the NE, *k*_diss_, an equilibrium constant for NE–GR_*n*_ association, β, and a concentration of interaction sites in the NE, Γ_S_. These parameters were defined by assuming that GR_*n*_ interacts with and translocates through the entire NE uniformly, i.e., the homogeneous model. The parameters based on the homogeneous model were converted to those based on the heterogeneous model, where GR_*n*_ interacts with and translocates through only the NPCs. The parameters based on both models are listed in Table 1. Recently, we validated both models for the interactions of the NPC with GR_20_, PR_20_, and protamine.^23^

Experimentally, NPC–GR_*n*_ interactions were assessed by measuring the transient current response of a micropipet tip positioned near and far from the NE. Initially, the potential of a micropipet was set positive enough not to transfer GR_*n*_ across the micropipet-supported interface from the aqueous solution to the NB solution. The potential of a micropipet was suddenly stepped negative enough at *t* = 0 to drive diffusion-limited GR_*n*_ transfer at the tip. The transient current response decayed gradually to eventually reach a steady-state value when the tip was positioned far from the NE, as illustrated for GR_25_ and GR_5_ (black lines in Figure 6A and 6B, respectively). A tip current response to GR_25_ was transiently higher when the tip was positioned near the NE, e.g., *d* = 0.5 μm (red line in Figure 6A). The higher current is attributed to the dissociation of GR_25_ from the NE and is emphasized when the tip current is plotted against 1/*t*^1/2^ (red circles in Figure 6A). The following steady-state current response to GR_25_ was lower when the tip was positioned near the NE, which hindered the diffusion of GR_25_ to the micropipet tip. By contrast, the transient current response of a micropipet to GR_5_ was only slightly higher when the tip was positioned near the NE (red circles in Figure 6B) owing to weaker NPC–GR_5_ interactions as confirmed below.

**Figure 7:**
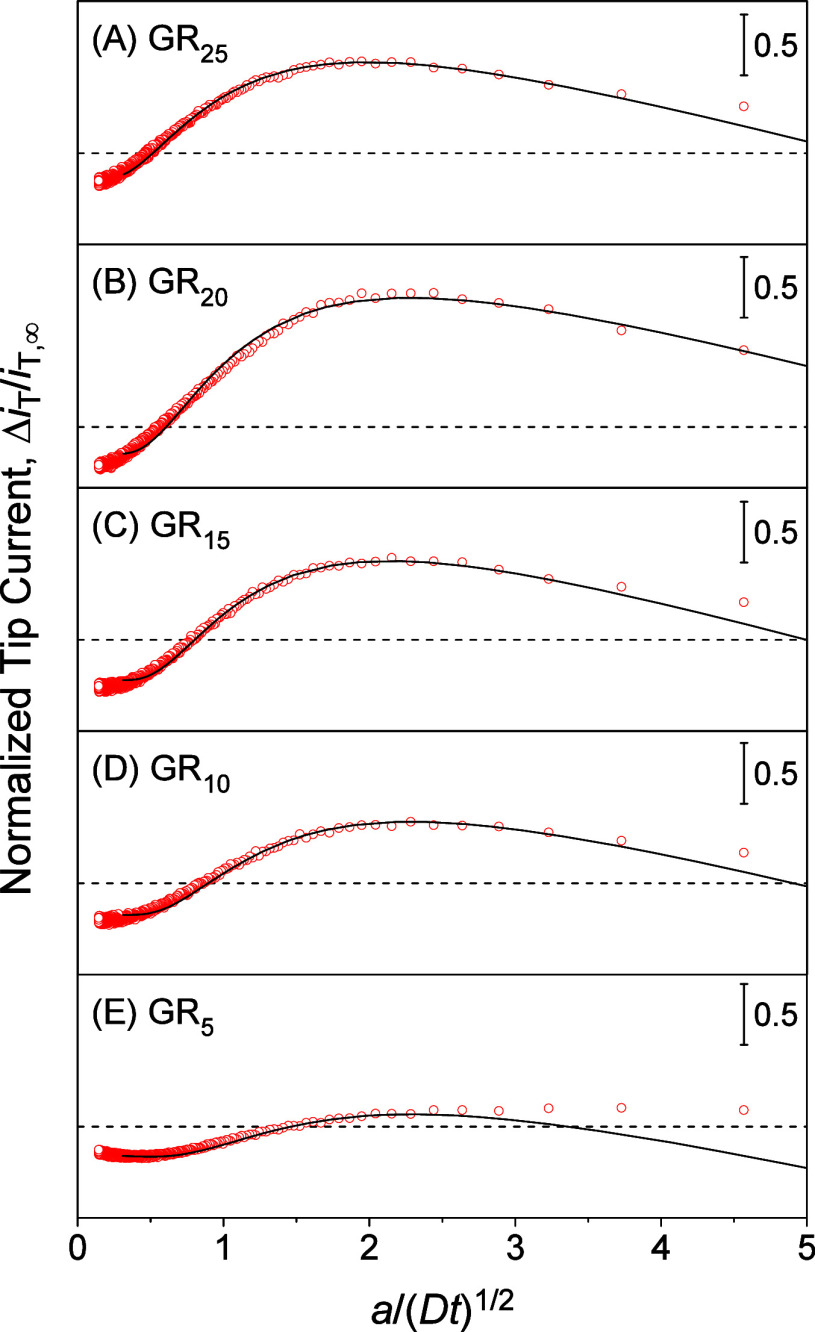
Experimental chronoamperograms of (A) GR_25_, (B) GR_20_, (C) GR_15_, (D) GR_10_, and (E) GR_5_ after subtraction (red circles) as obtained with 10 μm-diameter micropipets. The concentrations of the respective GR_*n*_ are 8.6, 10, 14, 21, and 130 μM in MIB. The current responses to the respective GR_*n*_ were fitted with theoretical ones (solid line) with the tip–NE distance of *d* = 0.5, 0.5, 0.25, 0.5, and 0.75 μm. Other parameters are listed in Table 1. Dashed lines represent zero current. Original chronoamperograms are shown in Figure 6 and Figure S2.

The time-dependent current response of a micropipet to GR_*n*_ was analyzed by the finite element method (Figure 7), which required the subtraction of nonfaradaic charging current from the experimental current response. The charging current was generated because the potential of a micropipet was stepped at *t* = 0 to polarize the micropipet-supported liquid/liquid interface. The charging current depends on the capacitance of the micropipet-supported liquid/liquid interface and the resistance between Ag and Ag/AgCl electrodes inside and outside of the micropipet, respectively (Figure 2).^49^ The resistance is dominated by the organic electrolyte solution in the micropipet and is independent of the tip–NE distance. Since the capacitance is also independent of the tip–NE distance, the charging current was eliminated by subtracting the chronoamperogram at the long tip–NE distance from that at the short distance.^50^ Good fits between experimental and simulated chronoamperograms after subtraction (Figure 7) confirm the elimination of the charging current. The subtracted current responses, Δ*i*_T_, are based on diffusion-limited GR_*n*_ transfer at the micropipet-supported interface and were simulated as reported elsewhere.^23^

### Quantitative Assessment of NPC–GR_*n*_ Interactions

Transient current responses of micropipets to GR_*n*_ after subtraction fitted with simulated current responses (Figure 7) to yield the interaction parameters based on the homogeneous model (Table 1). The finite element simulation was performed by using COMSOL Multiphysics, as reported elsewhere^23^ (also see the Supporting Information). The homogeneous model involves the association and dissociation of the entire NE with nearby GR_*n*_ as represented by the Langmuir-type isotherm. The NE associates more strongly with larger and more hydrophobic GR_*n*_ to yield larger β. Overall, interactions with the NE are ∼60 times stronger for GR_25_ than for GR_5_. In comparison with GR_25_ (Figure 7A), GR_5_ required a 15 times higher concentration to adsorb a sufficient amount of GR_5_ molecules on the NPC for the observation of a substantial transient current response (Figure 7E). Different concentrations of GR_*n*_ were considered in the finite element analysis of the transient response to determine β (see eq S9). By contrast, the smaller transient current response to smaller GR_*n*_ is enhanced by a higher dissociation rate constant and a higher concentration of interaction sites. Smaller GR_*n*_ can transfer into transport barriers more quickly and deeply to find more interaction sites, i.e., FG units (see below).

Stronger interactions of larger and more hydrophobic GR_*n*_ with the NE are mediated by the hydrophobic transport barriers of the NPC. Interaction parameters of GR_20_ are consistent with those of PR_20_, which is localized at the NPC, as confirmed by super-resolution fluorescence microscopy.^51^ The consistency excludes that GR_*n*_ interacts with or translocates through the outer and inner membranes of the NE. Moreover, the weak NPC–GR_5_ interactions were confirmed independently by atomic force microscopy (Figure S3 and Table S1). The NPCs were treated with GR_5_ to leave more central plugs than those with PR_20_, thereby ensuring that the latter interacted with FG units more strongly to replace the central plugs. The central plug is not intrinsic to the NPC and is an in-transit macromolecule trapped in the NPC through interactions with FG units.^52^

We converted the interaction parameters of the homogeneous model to those of the heterogeneous model (Table 1), where GR_*n*_ interacts with and translocates through the NPC only.^23^ Homogeneous and heterogeneous models are equivalent to each other thermodynamically as well as kinetically at steady states^22^ (see the Supporting Information). The thermodynamic equivalence is represented by the identical association constant, β, for homogeneous and heterogeneous models (eq S1). By contrast, interaction sites are localized in the NPC in the heterogeneous model to yield a higher concentration, Γ_S,NPC_, than Γ_S_ in the homogeneous model (eq S2). The resultant dissociation of GR_*n*_ from the more concentrated interaction sites in the heterogeneous model yields a lower rate constant, *k*_diss,NPC_, to maintain *k*_diss,NPC_Γ_S,NPC_ ≈ *k*_diss_Γ_S_ (eq S4). The residence time of a GR_*n*_ molecule at an FG unit (= 1/*k*_diss,NPC_ = 0.6–11 s from Table 1) is much longer than the residence time of an in-transit macromolecule in the NPC, i.e., <1 ms, for the transport of ∼1000 macromolecules per second,^53^ thereby clogging the nanopore for long to express neurotoxicity.

### Nanoscale Hydrophobicity of Transport Barriers

**Figure 8:**
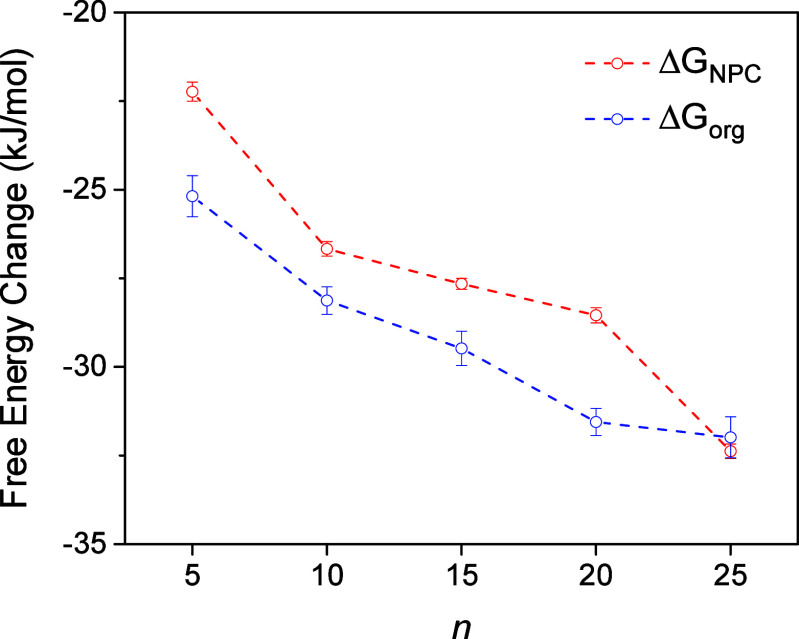
Length dependence of free energy changes in GR_*n*_ transfer at the NPC and the NB/water interface, Δ*G*_NPC_ and Δ*G*_org_, respectively.

We found that the free energy change in GR_*n*_ transfer from the aqueous solution to transport barriers in the NPC, i.e., Δ*G*_NPC_, becomes more negative to favor larger GR_*n*_ (Figure 8). This result confirms that the transport barrier of the NPC is a hydrophobic environment because larger GR_*n*_ is more hydrophobic.^19,20^ The free energy change is given by
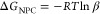
2

We also found that Δ*G*_NPC_ is more negative than the free energy of hydrophobic interactions as estimated for nuclear transport receptors. The standard free energy of −23.5 kJ/mol is estimated for the transfer of the physiological receptors into the hydrophobic condensate of FG-rich nups with partition coefficients of 1.3 × 10^4^.^12^ The more negative Δ*G*_NPC_ is attributed to cation−π interactions between the guanidinium unit of GR_*n*_ and the aromatic residue of an FG unit.^16,51^ The standard free energy of −2.9 ± 1.4 kJ/mol was estimated for cation−π interactions involving arginine in ∼2000 protein structures.^54^ The 1:1 binding of a GR_*n*_ molecule to an FG unit is also indicated by the agreement between the number of interaction sites and FG units in the NPC. The heterogeneous model yielded 10,000–26,000 sites per NPC (*N*_p_ in Table 1 and eq S3), which agrees with at least 13,000 FG units in each NPC.^55^

Interestingly, we also found that the free energy change in GR_*n*_ transfer from the aqueous solution to the NB solution, Δ*G*_org_, is very similar to Δ*G*_NPC_ (Figure 8). This similarity is supported by the similar relative permittivity of the water-saturated NB solution (ε_r_ = 35.5^30^) and the protein solution condensed by LLPS (ε_r_ = 30–60^31^). We estimated Δ*G*_org_ from a half-wave potential, *E*_1/2_ (Table 1), as
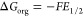
3

We defined Δ*G*_org_ as the free energy change per GR unit, i.e., *z* = +1, to compare GR_*n*_ with different charges. Since each guanidinium unit of GR_*n*_ is bound to the sulfonate group of a DNNS molecule electrostatically,^37^ Δ*G*_org_ is given by
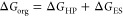
4where Δ*G*_HP_ is the free energy change in the transfer of a GR unit from the aqueous solution into the NB solution and Δ*G*_ES_ is the free energy change in electrostatic interactions of a GR unit with a DNNS molecule in the NB solution. In addition to hydrophobic interactions, Δ*G*_HP_ may include cation−π interactions between GR_*n*_ and NB molecules. We attribute the dependence of Δ*G*_org_ on the size of GR_*n*_ (Figure 8) to the size dependence of Δ*G*_HP_ owing to the consistency with the higher hydrophobicity of larger GR_*n*_.^19,20^ By contrast, Δ*G*_ES_ depends on the charge density of GR_*n*_ as predicted by the counterion condensation theory^56−58^ to weakly vary with the size of GR_*n*_ with the same charge density. Moreover, the contribution of Δ*G*_ES_ to Δ*G*_org_ is smaller with the NB solution than reported for the DCE solution^37,45,59^ with a lower ε_r_ of 10.1.^30^

We note that eq 3 neglects the electrochemical irreversibility of slow GR_*n*_ transfer at the NB/water interface (Figure 3), which shifts *E*_1/2_ more negatively to yield more positive Δ*G*_org_.^60^ This kinetic effect is larger for larger GR_*n*_ because the transfer of a larger ion is slower owing to complexation with more ionophore molecules at the liquid/liquid interface.^61^ Accordingly, more negative Δ*G*_org_ for larger GR_*n*_ is not due to the kinetic effect. By contrast, Δ*G*_NPC_ is purely thermodynamic without a kinetic effect, which is represented by *k*_diss_ (Table 1) to indicate the slower transfer of larger GR_*n*_.

## Conclusions

In this work, we employed GR_*n*_ as molecular probes to assess the hydrophobicity of transport barriers in the nanopore of the NPC. We confirmed stronger interactions of the NPC with larger and more hydrophobic GR_*n*_ as *n* increased from 5 to 25. The importance of hydrophobic interactions in NPC-mediated molecular transport has been hypothesized and examined by investigating the hydrogels of natural^12^ and mutated^13^ FG-rich nups and synthetic analogs.^14−16^ By contrast, we investigated the authentic NPCs of the nucleus isolated from the *X. laevis* oocyte to quantitatively demonstrate hydrophobic NPC–GR_*n*_ interactions. In addition, we found that larger GR_*n*_ stays at the NPC for longer, thereby expressing neurotoxicity.^17,18^ The interplay between thermodynamics and kinetics of NPC–GR_*n*_ interactions is highly significant to rationally design genetically therapeutic substances for nuclear import through the NPC. Our results imply that macromolecular and nanomaterial therapeutics for many genetic diseases^3^ must strongly associate with and rapidly dissociate from the NPC to enter the nucleus efficiently and nontoxically.

This work represents a powerful combination of transient SECM and liquid/liquid electrochemistry for the assessment of important biological hypotheses. Micropipet-supported liquid/liquid interfaces were employed as SECM tips to selectively detect GR_*n*_ with various sizes of up to *n* = 25 in the physiological buffer. Larger GR_*n*_ is more hydrophobic as expected theoretically^19,20^ and was more readily transferred into the NB phase by voltammetry. Interestingly, the free energy change in GR_*n*_ transfer at the liquid/liquid interface matched that at the NPC. This agreement indicates the broader and unexplored utility of the liquid/liquid interface as a model of hydrophobic biological environments beyond bilayer lipid membranes.^32^ Dielectrically, the water-saturated NB solution^30^ is more similar to LLPS-based protein condensates^31^ than to bilayer lipid membranes.^33^ LLPS is imperative biologically and biomedically^29^ but is not well understood,^62^ thereby requiring new experimental approaches.^63^ Our method will be useful to quantitatively investigate the interactions of molecules with LLPS-based proteinous media as exemplified by transport barriers in the NPC.^4^

## Supporting Information Available

SECM cell; chronoamperograms of GR_20_, GR_15_, and GR_10_; atomic force microscopy of NPCs; equivalence between homogeneous and heterogeneous models; finite element simulation; and details of COMSOL finite element simulation (PDF)
