# Supplementary material for: Nanoscale Hydrophobicity of Transport Barriers in the Nuclear Pore Complex as Compared with the Liquid/Liquid Interface by Scanning Electrochemical Microscopy
Source: Anal Chem. 2025 Jan 29;97(5):2745–53. doi: 10.1021/acs.analchem.4c04861 (PMC11822746; doi:10.1021/acs.analchem.4c04861)
Supplement: Supplementary file 1 — ac4c04861_si_001.pdf [file ac4c04861_si_001.pdf]

Supporting Information

Nanoscale Hydrophobicity of Transport Barriers in Nuclear Pore Complex as Compared with Liquid/Liquid Interface by Scanning Electrochemical Microscopy

Siao-Han Huang, Moghitha Parandhaman, Manu Jyothi Ravi, Donald C. Janda, and Shigeru Amemiya*

Department of Chemistry, University of Pittsburgh, 219 Parkman Avenue, Pittsburgh, Pennsylvania, 15260, United States

* To whom correspondence should be addressed. E-mail: amemiya@pitt.edu. Fax: 412-624-8611.

Contents:

1. SECM cell S2

2. Chronoamperograms of GR_{20}, GR_{15}, and GR_{10} S2

3. Atomic force microscopy of NPCs S4

4. Equivalence between homogeneous and heterogeneous models S5

5. Finite element simulation S6

6. References S7

SECM Cell. The NE was spread over a microporous membrane to serve as a substrate in the SECM cell (Figure S1) as detailed elsewhere.[S1]

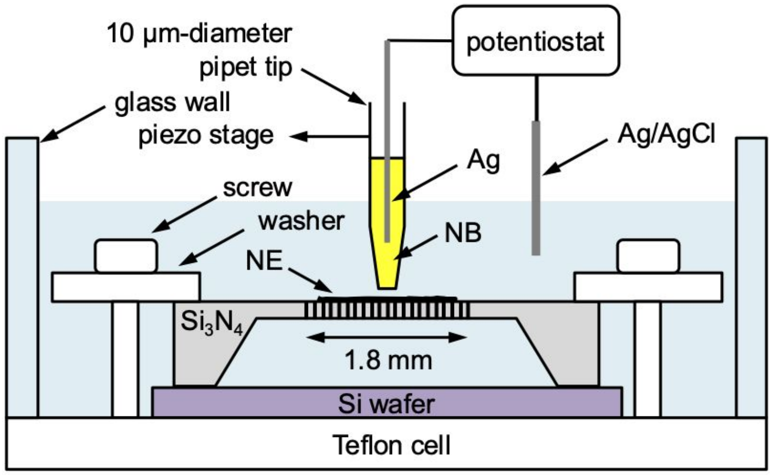

Figure S1. Scheme of SECM cell with the micropore-supported NE.

Chronoamperograms of GR_{20}, GR_{15}, and GR_{10}. A micropipet tip was positioned near and far from the NE to measure chronoamperograms of GR_n (n = 20, 15, and 10 in Figures S2A, S2B, and S2C, respectively). The tip current was plotted against time, t, or $1/\sqrt{t}$ to emphasize long-time and short-time behaviors. A chronoamperogram at the long tip–NE distance was subtracted from that at the short tip–NE distance. The subtracted chronoamperogram was compared with the theoretical one to determine interaction parameters (Figure 7).

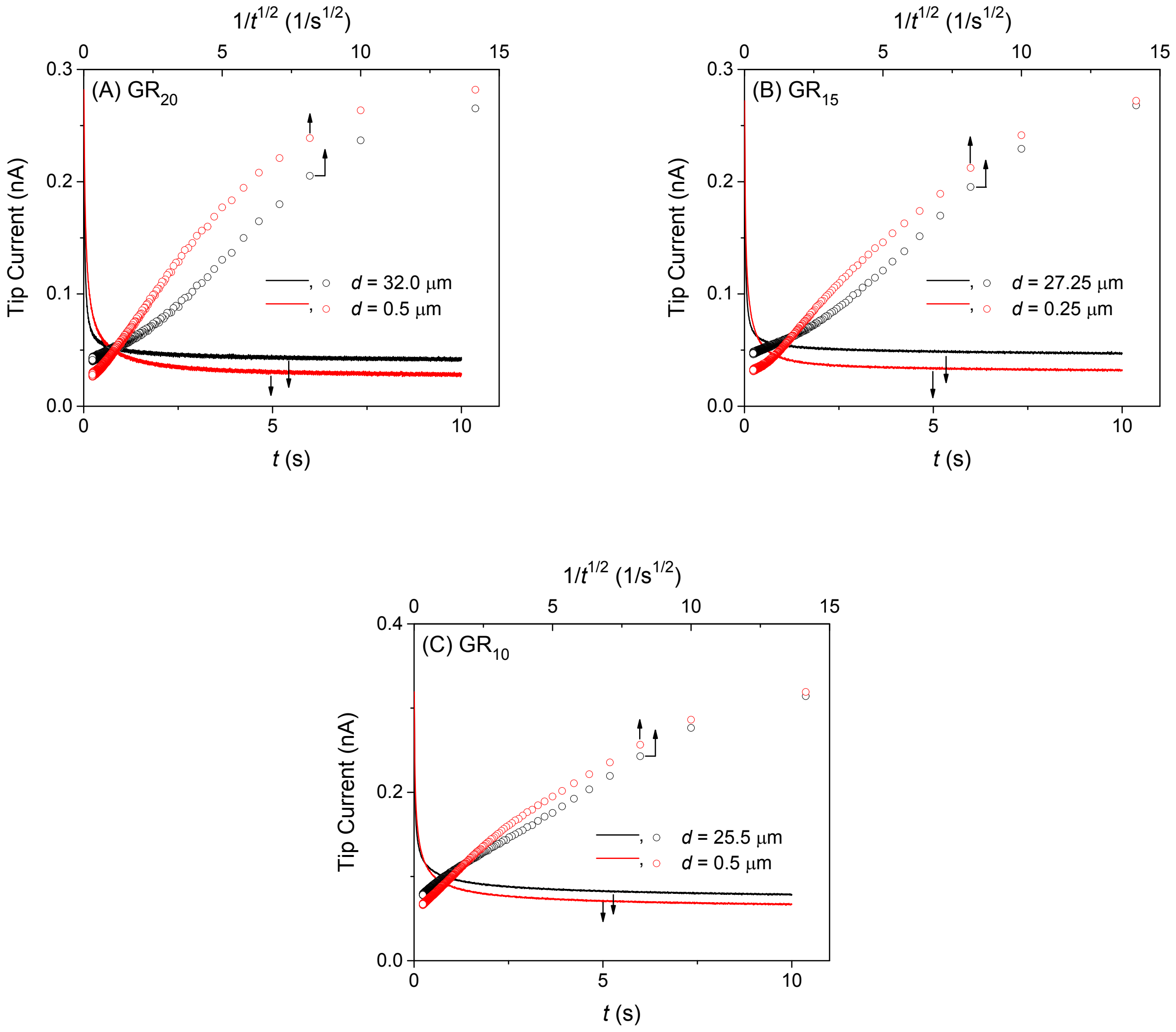

Figure S2. Chronoampergrams of (A) GR_{20}, (B) GR_{15} and (C) GR_{10} at 10 μm-diameter micropipet tips positioned far from (black) and near (red) the NE in MIB. Sampling interval, 5 ms. The tip–NE distance, *d*, was determined by the analysis of the chronoamperograms in Figure 7.

Atomic Force Microscopy of NPCs. We imaged the cytoplasmic side of the NE by atomic force microscopy (AFM) to find that the central plug of the NPC is hardly removed after being exposed to MIB containing GR_5. We assessed 53 NPCs treated with 130 μM GR_5 in the AFM image (Figure S3A) to find 24 recessed pores without a plug, 12 plugged pores, and 17 entangled pores as characterized by the cross sections of the NPCs in the AFM images[S1] (Figure S3B). The percentage of plugged NPCs is similar to that of the plugged NPCs treated with MIB only[S1] and significantly more than that of the plugged NPCs exposed to MIB containing PR_{20}, which strongly interacts with the FG units of the NPC to replace the central plug.[S6] By contrast, the interactions of GR_5 with the NPC were too weak to remove the central plugs but were still substantial to convert entangled NPCs to recessed NPCs in comparison with MIB only.

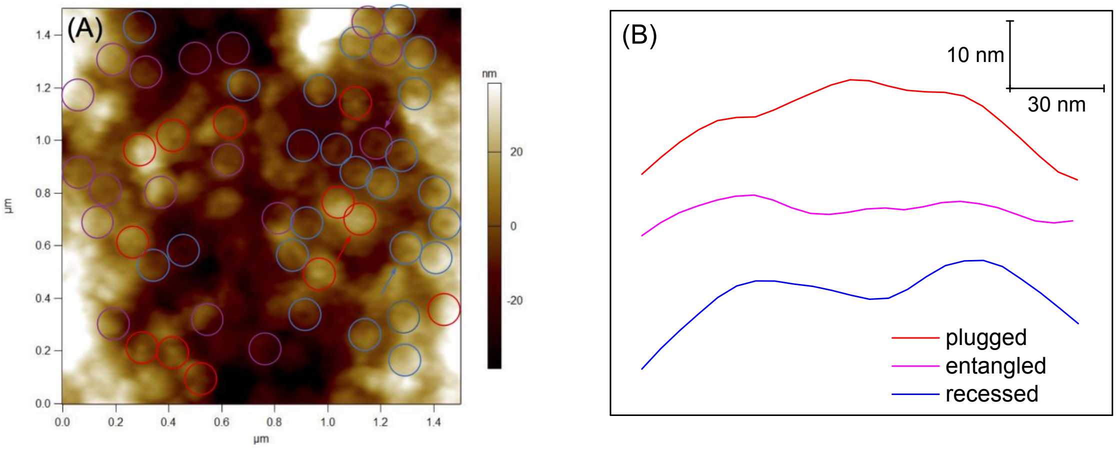

Figure S3. (A) AFM image of the cytoplasmic side of the NE treated with 130 μM GR_5 in MIB and fixed by glutaraldehyde. (B) Cross sections of plug-free recessed pore, plugged pore, and entangled pore. The corresponding pores are indicated by arrows in the AFM images. The NE was prepared and imaged as reported elsewhere.[S2]

Table S1. Percentages of Recessed, Plugged, and Entangled NPCs Determined by AFM.

	Recessed	Plugged	Entangled
130 μM GR_5 in MIB[a]	45 %	23 %	32 %
MIB only[b]	25 %	25 %	50 %
10 μM GR_{20} in MIB[c]	88 %	7 %	5 %

[a] From Figure S3A. [b] From ref. S1. [c] From ref. S6.

Equivalence between Homogeneous and Heterogeneous Models. Homogeneous and heterogeneous models are equivalent to each other thermodynamically as well as kinetically at steady states.[S3] The thermodynamic equivalence is represented by the identical association constant, β, for homogeneous and heterogeneous models as given by

$$\beta = \frac{k_{\text{ass}}}{k_{\text{diss}}} = \frac{k_{\text{ass, NPC}}}{k_{\text{diss, NPC}}} \qquad \text{(S1)}$$

where k_{ass} and $k_{\text{ass,NPC}}$ are association rate constants in homogeneous and heterogeneous models, respectively. Moreover, the total concentration of interaction sites must be identical between the two models to yield

$$\Gamma_{\text{s}} = \sigma\Gamma_{\text{s,NPC}} = \pi r^2 N\Gamma_{\text{s,NPC}} \qquad \text{(S2)}$$

where σ is the porosity of the NE, N is the NPC density, and r is the radius of the NPC nanopore. A σ value of 7.2×10^{-2} is estimated for the *Xenopus* oocyte nucleus with N = 40 NPCs/μm^2 and r = 25 nm.[S4,S5] Moreover, the number of interaction sites per NPC, N_{p}, was obtained from

$$N_{\text{p}} = \pi r^2 N_{\text{A}}\Gamma_{\text{s,NPC}} \qquad \text{(S3)}$$

where N_{A} is the Avogadro's number. We also estimated rate constants for the dissociation of GR_n from the NPC by employing the heterogeneous model as reported elsewhere[S6] to yield

$$k_{\mathrm{diss,NPC}} = \frac{k_{\mathrm{m}} k_{\mathrm{diss}} \sigma}{2\left(k_{\mathrm{m}} - \frac{k_{\mathrm{diss}} \beta \Gamma_{\mathrm{S}}}{2 + \beta c_0}\right)} \tag{S4}$$

with

$$k_{\mathrm{m}} = 4DNrf(\sigma) \tag{S5}$$

$$f(\sigma) = \frac{1 + 3.8\sigma^{5/4}}{1 - \sigma} \tag{S6}$$

where k_{m} (= 5.7×10^{-2} cm/s) is a rate constant for the mass transfer of GR_n near the NPC.

Finite Element Simulation. SECM-based approach curves and chronoamperograms were analyzed by using COMSOL Multiphysics (version 6.2, COMSOL, Inc., Burlington, MA) as reported elsewhere[S6] (also see the attachment for details). Steady-state NE permeability was determined from the analysis of approach curves. The simulation of chronoamperograms was performed to determine parameters for NE–GR_n interactions based on the homogeneous model. The interaction parameters were defined in the normalized forms as given by

$$\lambda = \frac{k_{\mathrm{diss}} a^2}{D} \tag{S7}$$

$$\kappa = \frac{\Gamma_{\mathrm{S}}}{a c_0} \tag{S8}$$

$$\rho = \beta c_0 \tag{S9}$$

where λ is the normalized dissociation constant, κ is the normalized concentration of interaction sites in the NE, and ρ is the normalized strength of NE–GR_n interactions. Eqs S7–S9 also considered the concentration, c_0, and diffusion coefficient, D, of GR_n as well as the inner radius, a, of a micropipet tip.

REFERENCES

(S1) Pathirathna, P.; Balla, R. J.; Jantz, D. T.; Kurapati, N.; Gramm, E. R.; Leonard, K. C.; Amemiya, S. *Anal. Chem.* **2019**, *91*, 5446.

(S2) Chen, R.; Pathirathna, P.; Balla, R. J.; Kim, J.; Amemiya, S. *Anal. Chem.* **2024**, *96*, 10765.

(S3) Huang, S.-H.; Amemiya, S. *Analyst* **2024**, *149*, 3115.

(S4) Eibauer, M.; Pellanda, M.; Turgay, Y.; Dubrovsky, A.; Wild, A.; Medalia, O. *Nat. Commun.* **2015**, *6*, 7532.

(S5) Jaggi, R. D.; Franco-Obregon, A.; Muhlhausser, P.; Thomas, F.; Kutay, U.; Ensslin, K. *Biophys. J.* **2003**, *84*, 665.

(S6) Huang, S.-H.; Parandhaman, M.; Jyothi Ravi, M.; Janda, D. C.; Amemiya, S. *Chem. Sci.* **2024**, *15*, 15639.
